# HIV Quasispecies Dynamics during Pro-Active Treatment Switching: Impact on Multi-Drug Resistance and Resistance Archiving in Latent Reservoirs

**DOI:** 10.1371/journal.pone.0018204

**Published:** 2011-03-24

**Authors:** Max von Kleist, Stephan Menz, Hartmut Stocker, Keikawus Arasteh, Christof Schütte, Wilhelm Huisinga

**Affiliations:** 1 Department of Mathematics and Computer Science, Freie Universität, Berlin, Germany; 2 Department of Gastroenterology and Infectious Diseases, Vivantes-Auguste-Viktoria-Klinikum, Berlin, Germany; 3 Hamilton Institute, National University of Ireland, Maynooth, Republic of Ireland; 4 Institute of Mathematics, University of Potsdam, Potsdam, Germany; British Columbia Centre for Excellence in HIV/AIDS, Canada

## Abstract

The human immunodeficiency virus (HIV) can be suppressed by highly active anti-retroviral therapy (HAART) in the majority of infected patients. Nevertheless, treatment interruptions inevitably result in viral rebounds from persistent, latently infected cells, necessitating lifelong treatment. Virological failure due to resistance development is a frequent event and the major threat to treatment success. Currently, it is recommended to change treatment after the confirmation of virological failure. However, at the moment virological failure is detected, drug resistant mutants already replicate in great numbers. They infect numerous cells, many of which will turn into latently infected cells. This pool of cells represents an archive of resistance, which has the potential of limiting future treatment options. The objective of this study was to design a treatment strategy for treatment-naive patients that decreases the likelihood of early treatment failure and preserves future treatment options. We propose to apply a single, pro-active treatment switch, following a period of treatment with an induction regimen. The main goal of the *induction regimen* is to decrease the abundance of randomly generated mutants that confer resistance to the *maintenance regimen*, thereby increasing subsequent treatment success. Treatment is switched before the overgrowth and archiving of mutant strains that carry resistance against the *induction regimen* and would limit its future re-use. *In silico* modelling shows that an optimal trade-off is achieved by switching treatment at 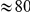 days after the initiation of antiviral therapy. Evaluation of the proposed treatment strategy demonstrated significant improvements in terms of resistance archiving and virological response, as compared to conventional HAART. While continuous pro-active treatment alternation improved the clinical outcome in a randomized trial, our results indicate that a similar improvement might also be reached after a single pro-active treatment switch. The clinical validity of this finding, however, remains to be shown by a corresponding trial.

## Introduction

In 1996, the tremendous clinical success of highly active antiretroviral therapy had led many researchers to believe that the eradication of HIV would be feasible. However, it was soon realized that inducible pro-virus persists in latently infected cells despite ongoing therapy and that the latent reservoir prevents HIV eradication within the patients lifetime [1]–[6].

Latent infection is established when 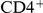 T-lymphoblasts containing integrated provirus [5], [7] escape both immune effector mechanisms and the cytopathic effects of the virus and revert to a resting memory state [8]. Besides preventing eradication of HIV, the latent reservoir also serves as a memory of any virus species replicating during the course of HIV infection [9], [10], including drug resistant variants. The contents of this archive of resistance are strong predictors of future treatment failure [9], [11].

Despite the impressive improvement of antiviral therapy, many patients still experience virological failure caused by the selection of drug resistant virus populations. Current guidelines recommend changing treatment after the confirmation of virological failure. However, in the face of the rapid viral turnover this approach could be sub-optimal [12]. Changing therapy after the appearance of drug resistant mutants will (i) allow the resistant viral population size to expand and evolve and (ii) lead to an archivation of resistant viral strains. An optimal treatment strategy should therefore prevent viral relapse with drug resistant strains and, more importantly, prevent drug resistant mutants from establishing latent infection.

Induction-maintenance (IM) approaches are used for the treatment of a growing number of infectious- and neoplastic diseases [13]–[15]. Typically, patients start with an intensified induction regimen (composed of a number of potent and potentially toxic drugs), which will subsequently be replaced by a maintenance regimen (composed of a smaller number of less toxic drugs) [16]. However, patients treated with a large number of drugs are particularly vulnerable to drug interactions [17] and adverse side effects that complicate HIV therapy and seriously undermine the success of clinical management [18].

Another approach to overcome the development of resistance is to alternate antiretroviral therapy [19]. This strategy has been shown to significantly delay virological failure [20], [21], yet it is flawed by its high psychological and physical burden [22].

We propose an approach that combines the advantages of conventional IM- and treatment alternation strategies, but minimizes their inherent disadvantages. We suggest a single, pro-active treatment switch from an inducer drug combination to a maintenance combination. The inducer drug combination should rapidly lower the viral population size and eliminate resistant mutants. Subsequently, it will be replaced by a maintenance drug regimen with a completely different resistance profile, before drug resistant strains are archived.

We have previously introduced a novel model of virus dynamics and adaptation [23], which allows us to consider the distinct molecular effects of all novel (and some developmental) HIV drugs. In this article, we present a novel mathematical concept, which prevents the emergence of drug resistance in each individual realization (virtual patient) of the model by switching between therapies. Utilizing this concept, we deduce a distribution of (individual) switching-times, which we use to determine a single fixed duration for the induction therapy, which increases the treatment success probability in the whole virtual patient population and which minimizes the risk for resistance to become archived in the latent reservoir. Finally, the performance of this novel induction-maintenance-strategy is evaluated against conventional HAART therapy.

## Results

### Virus dynamics model

**Figure 1:**
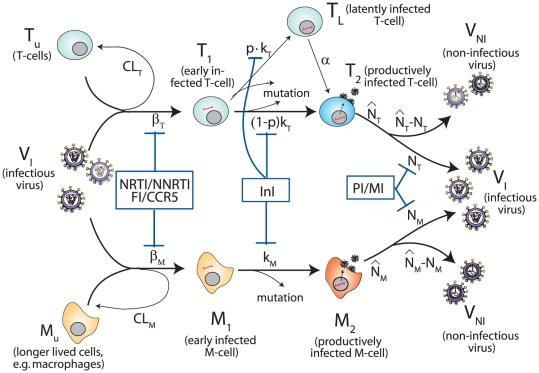
Extended virus dynamics-, mutation- and drug interference model. Target cells (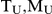) can become successfully infected by infective virus 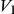 with infection rate constants 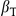 and 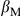, respectively, creating early infected cells 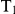 and 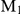. Infection can also be unsuccessful after the step of viral fusion (rate constant 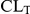 and 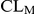), eliminating the virus and rendering the cell uninfected. Early infected cells 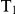 and 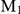 can also destroy essential viral proteins or DNA prior to integration, returning the cell to an uninfected stage. The genomic viral DNA can become integrated with rate constants 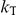 and 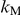 creating post-integration, infected cells 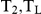 and 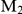. The latently infected cell type 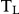 does not express viral genes, but can become activated with rate 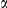, transforming this cell into a productively infected T-cell 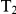. Virus producing cells 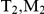 release new infectious- and non infectious virus 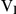 and 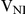 with rate constants 
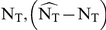
 and 
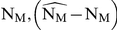
, respectively. Phenotypic mutation occurs at the stage of viral genomic integration 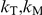 (see [23]). All cellular compartments 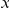 can get destroyed by the immune system with respective rate constants 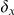 and the free virus (infectious and non-infectious) gets cleared with rate constant 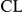 (not shown in the illustration). The site of drug interference with the replicative cycle of HIV is indicated by blue bars for the respective drug classes (NRTIs, NNRTIs, FIs, CCR5-inhibitors, INIs, PIs, and maturation inhibitors).

We have extended the existing viral dynamics model, described in [23], for the compartment of very long lived, latently infected T-cells 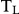 (Fig. 1 and *Materials and Methods* section), which are believed to prevent eradication of HIV [24] and to lead to the archiving of drug resistance [9], [10].

Briefly, the virus dynamics model (Fig. 1) comprises T-cells, macrophages, free non-infectious virus (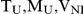, respectively), free infectious virus of mutant strain 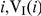, and five types of infected cells belonging to mutant strain 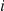: infected T-cells and macrophages *prior* to proviral genomic integration (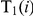 and 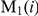, respectively) and infected T-cells and macrophages *after* proviral genomic integration (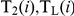 and 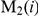, respectively). The latently infected cell type 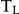 does not express viral genes, but can become activated with rate 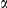, transforming this cell into a virus producing post-integration infected T-cell 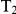. The average rates of change of the different species are displayed in the *Materials and Methods* section. All parameter values have been chosen according to previous studies and are displayed in Table 1. Since some viral strains are present only in very low copy numbers, we used a hybrid stochastic-deterministic approach [25] to perform simulations (see *Materials and Methods* section for details).

**Table 1:** Model parameters generally used in simulations.

Param.	Value	Ref.	Param.	Value	Ref.
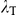	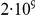	[64]	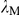	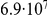	[65]
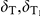	0.02	[65]	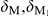	0.0069	[65]
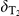	1	[36]	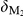	0.09	[23]
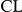	23	[36]	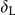	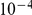	[16], [66]
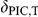	0.35	[67], [68]	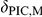	0.0035	[23]
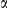	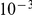	[66]	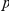	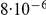	[66]
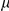	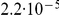	[42]	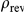	0.33	[68], [69]
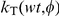	0.35	[68]	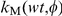	0.07	[23]
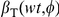	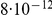	[49]	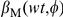	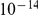	[23]
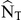	1000	[65]	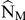	100	[65]
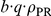	0.67	[23]	-	-	-

All parameters refer to the wildtype 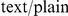 in the absence of drug treatment 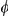. All parameters in units [1/day], except 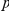, 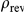, 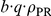 (unit less) and 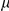 in 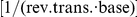. 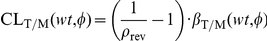, 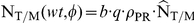 [23].

### Treatment change before virological failure

**Figure 2:**
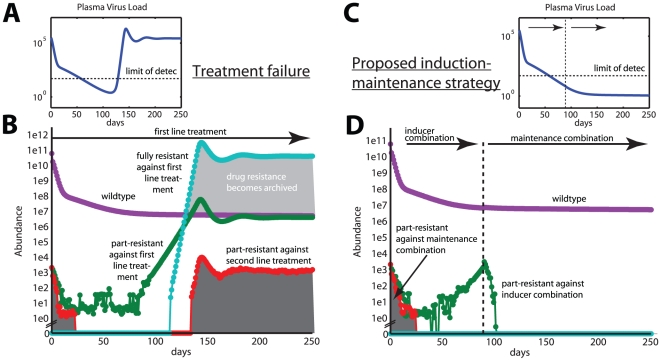
Abundance of viral mutants during first-line treatment failure and during proposed induction-maintenance strategy. A: Plasma virus load during first line treatment failure (blue line). B: Total abundance of distinct viral mutants during first-line treatment failure. C: Plasma virus load (blue line) during proposed induction-maintenance strategy with switch between induction- and maintenance treatment at 80 days (vertical dashed line). D: Total abundance of distinct viral mutants during proposed induction-maintenance strategy. The magenta line denotes the abundance of wildtype virus. Green- and cyan lines denote the abundance of mutants that are part-resistant against the first line regimen (resistant against two out of three drugs) and mutants that are fully resistant against the first line regimen, respectively. The red lines denote the abundance of all mutants, which are part-resistant against a second line treatment. The area under the red line is highlighted by the dark grey shaded area, to stress the negative impact of these mutants on the success of a second line regimen. The light shaded area in panel B indicates that resistant mutants are more abundant than the wildtype and therefore highlights when drug resistance archiving in latently infected cells takes place. The simulations were performed by assuming 70% drug efficacy 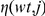 and a fitness loss 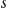 of 20% per drug resistance mutation. Furthermore, it was assumed that a single point mutation can confer absolute resistance to a single drug.

Currently, changes of antiretroviral treatment regimes are largely triggered by virological failure or toxicity. In Fig. 2A, we show the simulated viral load in the case of first line treatment failure. The corresponding population dynamics of HIV are shown in Fig. 2B. During first line treatment failure, resistant mutants (green- and cyan colored lines in Fig. 2B) are selected from the quasi-species population and quickly evolve into the dominant virus population, leading to viral rebound. While the total virus population is temporarily shrinking, mutants that confer resistance against a potential follow-up treatment (red line, dark grey shaded area in Fig. 2B) are depleted (possibly eradicated). However, during viral rebound the total viral population re-expands and consequently erroneous reverse transcription generates novel mutants that can confer resistance against a second line therapy. Once the viral population size has been restored, the second line therapy, although composed of entirely different drugs, is as likely to fail as before the initiation of first line therapy. Furthermore, it is likely that drug resistant viral strains become archived while they dominate the viral population (light grey shaded area in Fig. 2B).

In Fig. 2C, we show the viral load dynamics during the proposed induction-maintenance therapy. The corresponding population dynamics of HIV are shown in Fig. 2D. The inducer combination reduces the viral load (see Fig. 2C). However, treatment is changed (vertical dashed black line) to the maintenance combination, before resistant strains (green and cyan line in Fig. 2D) can become more abundant than the wildtype (magenta line in Fig. 2D). Therefore, at the time of treatment change (vertical dashed black line in Fig. 2D), total virus has been decreased and mutants that confer resistance to the maintenance therapy (red line, dark grey shaded area in Fig. 2D) are likely to be eradicated, which improves the probability to achieve durable virological suppression with the maintenance therapy. With this strategy, the abundance of the wildtype is larger than the abundance of drug-resistant mutants, which lowers the probability that drug resistance enters the latent reservoir (light grey shaded area is absent in Fig. 2D).

In order to determine the optimal time point for switching from inducer- to maintenance- drug combinations, 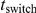, we first determined relevant sets of parameters for (i) the *in vivo* efficacy 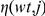 of each utilized drug 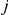 against the wildtype 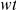 and (ii) the *in vivo* fitness loss that is associated with resistance development 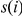 (shown in Table S1), since the corresponding *in vivo* parameters are known to vary substantially between different patients, e.g. [26]. For simulation purposes, we assumed that a single point mutation is sufficient to create high-level resistance (99%) to a single drug. This is somewhat a worst-case assumption, but is justified for a number of drugs, see e.g. [27], [28]. Relevant clinical failure rates after one year in previously treatment-naive patients, who receive HAART in a clinical trial setting, are 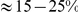 [29], (see Table S1).

**Figure 3:**
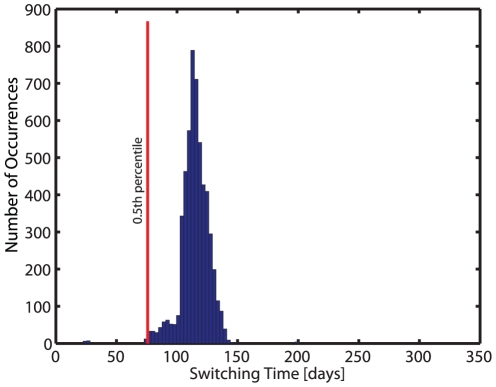
Histogram of optimal, individual treatment switching times. Switching times for changing from inducer- to maintenance therapy were automatically determined and carried out (using eq. (1)). The 0.5th percentile, marked by the red line, was determined and the corresponding time 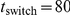 days was used as a fixed value in the suggested strategy to switch from inducer- to maintenance therapy. Hybrid deterministic-stochastic simulations were performed at clinically relevant parameter sets (see Table S1). Drug switches occurred in a total of 5478 out of 6000 simulations.

We then use an algorithm that automatically switches from inducer- to maintenance drug combination, minimizing virological failure for each realization (virtual patient), respectively. A histogram of the derived (individual) switching times from a total of 6000 simulations is shown in Fig. 3. Based on the histogram, we finally chose a fixed time 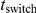 for changing from induction- to maintenance therapy. In the sequel, we evaluate, if the chosen time 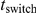 to change from inducer- to maintenance combination leads to a general improvement compared to conventional HAART therapy, in terms of treatment success and drug resistance archiving.

### Determination of treatment changing time

In [23] we introduced the ‘reproductive capacity’ 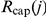. For the extended model used herein, we have provided the derivation of 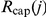 in the *Materials and Methods* section. The reproductive capacity 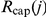 can be envisaged as the amount of offspring that the whole viral population is expected to produce under some treatment 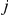 during one round of replication. It can be calculated from any model simulation and enables to evaluate each state of the infection from the perspective of any potential treatment 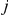. As the viral population adapts to some currently applied treatment, 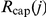 changes accordingly: 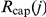 is large initially and decreases subsequently until drug resistant strains develop and render the treatment 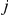 inefficient. We want to assess the point in time, when some inducer- drug combination stops to provide any benefits (in terms of the viral population) for the *next* drug combination (maintenance combination). We therefore evaluate 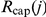 for 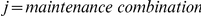 while the induction combination is applied and change from the induction- to the maintenance therapy when 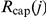 reaches its minimum;
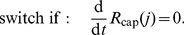
(1)


The derived switch-times are displayed in Fig. 3. We chose the 0.5th percentile at 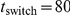 days as a fixed time for treatment change in the forthcoming evaluation of the proposed induction-maintenance-strategy.

### Implementation of conventional vs. proposed induction-maintenance-strategy

In order to reflect the clinical practice of HIV care, we have implemented the following routine for assessing the efficacy of the applied treatment combinations.

Our virtual patients are monitored every month for efficacy assessment until virus levels fall below the limit of detection (50 HIV RNA/mL plasma). Thereafter, they are monitored every other month. Virological failure has been defined according to treatment guidelines [24]: At the first efficacy assessment (one month after treatment initiation), viral load should have fallen by at least 2 logs [HIV RNA/mL plasma]. Each consecutive measurement should be below the previous assessment. By month 4, viral load should be below the level of detection (50 HIV RNA/mL plasma). After that, detectable virus is defined as virological failure.

We implemented conventional HAART in the following way: The virtual patients are initially treated with a drug combination consisting of two nucleoside reverse transcriptase inhibitors (NRTIs) and one non-nucleoside reverse transcriptase inhibitor (NNRTI) (e.g. tenofovir (TDF) + emtricitabine (FTC) + efavirenz (EFV)), until virological failure is detected, in which case treatment is changed to a second line regimen consisting of a protease inhibitor (PI), an integrase inhibitor (InI) and an entry inhibitor (EI) (e.g. ritonavir (RTV) -boosted PI + raltegravir (RLV) + maraviroc (MVR)).

In the proposed induction-maintenance-strategy, patients are initially treated with a combination consisting of a PI, an InI and an EI, until 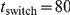 days. After that, a treatment consisting of two NRTIs and one NNRTI is applied. If failure is detected at any efficacy assessment time point, treatment change is applied.

In the following, we performed 1000 hybrid stochastic-deterministic simulations for each relevant parameter set (deduced from Table S1) and counted the number of realizations, in which virological failure occurred. Furthermore, we assessed, if the number of drug resistant mutants in the very long-lived infected cells 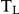 was higher at the end of the simulation than upon initiation of treatment. In this case we recorded “archiving” of drug resistance. The results of our simulations are discussed in the next section.

### Proposed induction-maintenance-strategy improves success rate and minimizes archiving of drug-resistance

**Figure 4:**
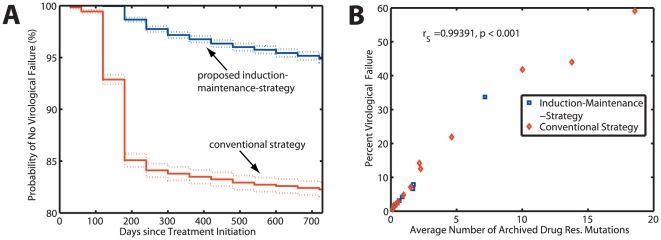
Kaplan-Meier estimates for treatment success, and correlation between virological failure and archiving of drug resistance. The plots summarize the results trough the whole simulated parameter space from Table 2 (12000 simulations in total). A: Probability of no virological failure (%) for the IM-strategy (blue line) and the conventional therapy (red line), respectively. Dashed lines are the 95% confidence ranges, calculated using Greenwood's formula. Virological failure was defined according to [24] and is summarized in section “Implementation of conventional vs. proposed induction-maintenance-strategy”. B: The probability to virological failure vs. the average number of drug resistance archiving in the latent reservoir. A strong positive correlation (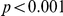) between virological failure and drug resistance archiving exists, as indicated by spearman's non-parametric rank correlation coefficient 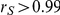.

Fig. 4A shows that the proposed induction-maintenance-strategy (blue line) with a fixed treatment switch time of 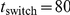 days leads to a significant reduction in the probability to experience virological failure compared to the conventional treatment strategy (red line). This observation holds true for a wide range of parameters (see Table 2, second column). In only two cases, where failure rarely occurs during conventional therapy, we do not get significant differences at the p = 0.05 level.

**Table 2:** Probability of virological failure and -archivation of multi-drug resistant virus during suggested induction-maintenance- (IM) vs. conventional HAART strategy.

Parameter set	Failure rate	Probability of multi-drug resistance archivation		
ID ( 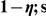 )	IM, HAART	≥2 mutations	≥3 mutations	≥5 mutations
R1 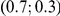	1.7, 4.8% 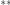	1.8,4.8% 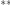	1.7,4.8% 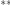	0,0.1%
R2 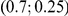	4.2, 14.2% 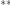	4.8,14.2% 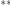	4.2,13.9% 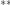	0.1,0.2%
R3 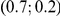	6.6, 41.8% 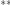	18.5,42.2% 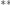	9.6,41.6% 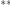	0.1,2.9% 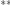
R4 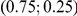	0.9, 2.8% 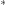	0.9,2.9% 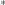	0.9,2.8% 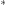	0,0%
R5 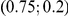	1.8, 12.5% 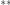	2.2,12.6% 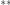	1.8,12.5% 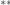	0,0.4%
R6 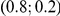	0.7, 2.2% 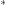	0.8,2.3% 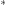	0.7,2.2% 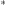	0,0.2%
R7 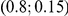	3.1, 21.9% 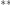	2.8,22.1% 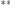	3.1,21.9% 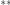	0.2,0.9% 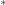
R8 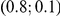	7.9, 44% 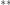	9.3,44% 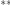	8.3,44% 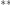	0.7,14.6% 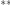
R9 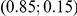	0.6, 0.6%	0.9,1.3%	0.6,0.6%	0,0%
R10 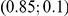	2.4,7.1% 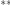	2.7,8.1% 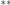	2.4,7.2% 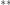	0.3,0.4%
R11 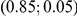	33.7, 59.1% 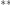	34.7,59.5% 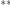	34,59.3% 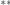	3.4,17.2% 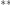
R12 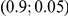	1.2, 1.8%	2.3,2.5%	1.3,1.8%	0.1,0.1%

Columns 2–5 show the distinct treatment outcome for the suggested induction-maintenance strategy (left entry) and a conventional HAART strategy (right entry) for different parameter sets R1–R12 in terms of mutation-associated reproductive fitness losses 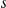 and different levels of drug efficacy 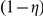 (indicated in column 1), following 1000 simulations respectively. Relevant parameter combinations had been identified beforehand, see Table S1 and section “Treatment change before virological failure”. Column 2: Percentage of virological failure after 2 years of therapy according to the HIV treatment guidelines (summarized in section “Implementation of conventional vs. suggested induction-maintenance strategy”). Column 3–5: Probability of multi-drug resistance archiving during the proposed strategy and during conventional HAART strategy. Cross tab 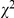 tests of independence between treatment strategy (suggested vs. conventional strategy) and outcome (virological failure or archivation of multi-drug resistance) are stated. A small 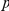-value indicates that the distinct outcome depends on the treatment strategy and is not due to random effects (** 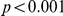, * 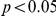).

Fig. 4B shows that virological failure and the average number of archived drug resistance mutations are strongly correlated (spearman's correlation coefficient 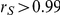, 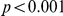). This indicates that virological failure is a strong predictor for drug resistance archiving.

Tables 2 (third–fifth column) show the number of cases in which archiving of multi-drug resistant viral strains (with 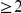, 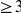 and 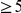 drug resistance mutations) occurred in the latent reservoir, under the proposed induction-maintenance strategy and conventional HAART, respectively. It can be seen that the proposed treatment strategy leads to a significant reduction in multi-drug resistance archiving for the majority of parameters evaluated. This indicates, that although two treatment lines have been used for the novel therapy, more therapeutic options are on average available in the follow-up period, compared to conventional therapy.

## Discussion

We have presented and tested (in terms of a mathematical model) a very simple treatment strategy that can lead to significant reductions in virological failure in comparison to conventional HAART treatment. A unique drug combination (inducer combination) is used for a short time (80 days) and pro-actively switched to a maintenance combination. The purpose of the inducer combination is to decrease viral population size and thereby increase the likelihood that the subsequent therapy (maintenance) will achieve durable suppression. Clinical implementation of this novel treatment strategy requires only one additional clinical visit at 80 days in comparison with the conventional HAART therapy. The important finding of our study is, that although two drug combinations are always utilized during the proposed induction-maintenance strategy, less archiving of drug resistance occurs in comparison with a conventional treatment strategy, where a second treatment line would be applied only in the case of virological failure or toxicity. Less drug resistance archiving implies that more treatment options will be available for the follow-up and long-term management of HIV-infected patients when the proposed induction-maintenance treatment strategy is used (see Table 2, third–fifth column).

Fig. S1 shows that only a few archiving events (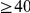 fully resistant mutants) are sufficient to eliminate treatment options permanently. The number of circulating latently infected cells is small [2], [7], [30], [31]. Detecting a small subset of mutants within the circulating latently infected cells is experimentally not feasible, because standard sequencing technology will detect the major strains [32], while novel, second generation methods require large samples [33]. Hence, mathematical modelling is a reasonable tool to investigate drug resistance archiving following treatment application.

The time for switching between combinations 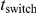 ( = 80 days) is the most critical parameter for the success of the proposed strategy. The following two considerations have to be taken into account: (i) The inducer combination should be applied only for a short time, to prevent the selection and archiving of mutants, which are resistant to the *current* drug combination and would limit the further use of this drug combination (risk of the strategy), (ii) while at the same time, it has to be applied long enough to possibly eradicate viral mutants, which are resistant to the *next* drug combination (the benefit of the strategy).

**Figure 5:**
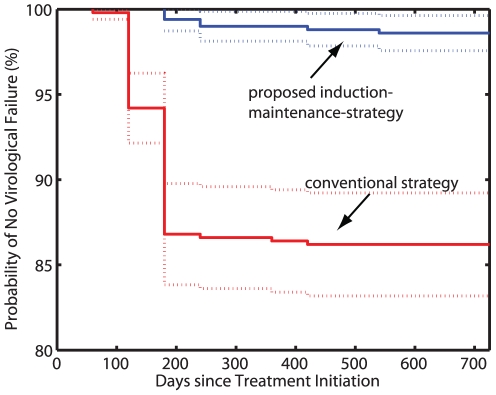
Kaplan-Meier estimates of treatment success (probability of no virological failure) for very high initial abundance of drug resistant mutants. The figure shows the outcome of 500 simulations for the proposed induction-maintenance strategy (blue line) and for the conventional HAART therapy (red line), respectively. Dashed lines indicate the 95% confidence ranges, calculated using Greenwood's formula. The initial abundance of drug resistant mutants was set to 1% of the population. Other parameter values: 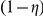 = 0.75, 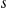 = 0.8.

The time required for resistant mutants to emerge, depends on their abundance before the initiation of therapy (if they pre-exist and are selected from the population) and also on their genetic distance to the wildtype (if resistance is *de novo* developed). As discussed above, we determine the abundance of mutants at the time of therapy initiation by utilizing the deterministic fix-point as starting condition for our simulations. We have shown the non-inferiority of our approach in Fig. 5, if drug resistant mutants are more abundant than expected. We have assumed the shortest genetic distance possible between wildtype and fully drug resistant mutants (one mutation is sufficient to create full resistance against a single drug, three distinct mutations are required for full resistance against a triple-drug combination). For some drugs, however, subsequent accumulation of mutations creates fully drug resistant mutants [34]. In our model, drug resistance might therefore develop more rapidly than *in vivo* for drugs with a large genetic barrier [35]. This implies that *in vivo* the inducer combination could possibly be applied for a longer time frame than the 80 days utilized in our model, if the genetic distance between wildtype and fully drug resistant mutant was greater than considered here (greater than one point mutation). However, our results demonstrate that even a very short time (80 days) in which the inducer combination is applied, can improve the clinical outcome significantly (see Fig. 4 and Table 2). This short time already minimizes the probability that drug resistance emerges and can, in that sense, be considered safer than a longer induction phase.

Eradication of viral mutants depends critically on their abundance prior to the initiation of therapy and on the rate at which viral compartments (and therefore resistant mutants) are cleared *in vivo*. The elimination of viral compartments *in vivo* has been quantified and validated in a number of clinical studies [36]–[38]. We used the expected abundance of viral mutants (the deterministic fix-point of the model) to estimate the abundance of different viral mutants at the time of treatment initiation. In Fig. 5 we show non-inferiority of our approach in the case, where an unexpectedly high abundance of drug resistant mutants is present (1% of the wildtype; detection limit of second generation sequencing technologies [33], [39], [40]), which would require longer time for eradication.

One limitation of the proposed induction-maintenance strategy is the potential inability to eliminate viral strains, that carry resistance to the maintenance therapy. This is particularly the case, if viral mutants, which carry resistance against all (or at least the majority of) drugs in the maintenance combination, are archived in the latent reservoir prior to treatment initiation. In Fig. S1B, we have quantified that 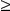 40 fully resistant viral mutants in the latent reservoir eliminate treatment options permanently. However, the likelihood for fully resistant archival copies (resistant against all drugs in the maintenance regimen) in the treatment naive patient, who was infected with wildtype 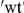 virus, is relatively small. Based on quasi-species theory, Ribero et al. [41] calculated the pre-treatment frequency of viral mutants. According to [41], the frequency of double mutants (part-resistant) relative to the wildtype equals
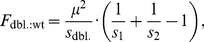
(2)where 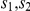 and 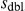 are the selective disadvantages of the strain carrying the first-, the second- and the both drug-resistance mutations and 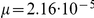 is the single point mutation rate [42]. It is reasonable to assume that resistant mutants are, at best, as likely to enter the latent reservoir as the wildtype in the absence of any drugs, due to their inherent fitness loss, i.e. 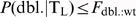. Considering a maintenance combination consisting of efavirenz (EFV), tenofovir (TDF) and emtricitabine (FTC), with primary resistance mutations K103N, K65R and M184V and respective selective disadvantages for the single-point mutants 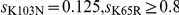 and 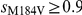 [43] and additive fitness losses in the double mutants 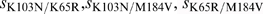 (i.e. 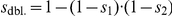), the probability that mutants, resistant against two out of three maintenance drugs, enter the latent reservoir are 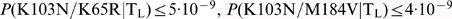 and 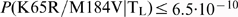 respectively. Using *in vivo* data, Chun et al. [7] estimated the average number of latently infected cells with replication-competent provirus to be 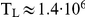 cells, so that the expected number of partly-resistant mutants 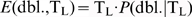 that are archived prior to treatment initiation is 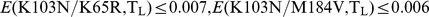 and 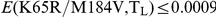. In other words, it is very unlikely that part-resistant mutants are archived in patients prior to treatment, since 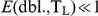. Furthermore, part-resistant mutants are still susceptible to one out of the three drugs in the maintenance combination. For triple-drug (fully) resistant strains, the likelihood of archival copies is even smaller.

Infection with drug resistant strains, mainly against established drug classes, is a major, growing health concern [44]. During infection with drug-resistant viral strains, archivation in the latent reservoir is likely, since this reservoir is established early in the infection [45]. If the circulating viral population reverses to a drug-susceptible type, archived resistant mutants from the time of infection might remain undetected and can complicate subsequent treatment (see Fig. S1). This particular circumstance applies equally to the proposed induction-maintenance therapy and conventional HAART.

For our strategy, we have chosen drugs from novel classes (e.g. InI, EI) for the inducer-combination, while we selected drugs from well-established classes for the maintenance combination (NNRTI, NRTI). This has the following rationale: The inducer combination will only be applied for a short time (80 days), while the maintenance combination could possibly be applied for much longer periods of time (until it fails, or toxicological events occur). Second or third generation drugs within the established drug-classes are often more convenient to apply (e.g. once daily dosing) and are less toxic, which has important implications for the long-term management of HIV [46]. Secondly, drugs from the novel drug classes (InI, EI), are currently not available as generic formulations, whereas low-cost alternative drugs exist for established drug classes. Therefore, in order to reduce treatment costs, it is of advantage to select a strategy, in which inexpensive drugs can be used for the majority of time, while cost-intensive ones are only applied for short treatment periods.

Some drug classes can cause a distinct viral load decline. In particular, the only approved InI raltegravir causes a more rapid viral load decay, compared with other HIV inhibitors [47], [48]. It might therefore seem logical, based on viral load decay, to use raltegravir in the induction treatment. It has been shown, however, that the faster viral decay with raltegravir could be a consequence of the particular site of action of InIs within the viral life cycle and may not be due to an overall increased removal rate of replication-competent viral compartments by raltegravir [23], [49]. Long-term studies of raltegravir- versus efavirenz-based HAART showed equal outcomes with either therapies [50], [51], arguing against the superiority of raltegravir-based drug combinations in removing replication-competent virus; however, further analysis is required.

Intuitively, it might be more advantageous to use drug resistance tests to guide treatment switches, instead of using a fixed time for a pro-active switch from inducer- to maintenance combination [19]. However, under the considerations discussed above, a switch from inducer- to maintenance combination should be applied before any resistant strains become abundant. This implies that the most frequent viral strain at the time of switch should be the wildtype. Standard assays fail to detect minority species [32]. Ultra-deep/pyro-sequencing might provide a more holistic picture of the quasi-species composition and can pick up viral mutants that are abundant in 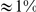 of the quasi-species population and if the sample is large enough [33], [39], [40]. However, even in this case, viral mutants are likely to dominate once the results are available (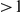 week), owing to the rapid viral kinetics [52].

In our *in silico* study, we considered time-invariant, as well as anatomically homogeneous *average* drug efficacy 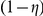, for the ease of modelling. It is also possible to consider drug- and patient-specific time-varying pharmacokinetics and to study the impact of compliance on drug resistance development. However, if compliance is identical between the two study arms, the qualitative difference between the outcome of conventional HAART versus the proposed induction-maintenance strategy is not expected to change. As shown in Table 2, the proposed induction-maintenance therapy performs better than conventional HAART for a wide range of parameter values for 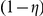. Furthermore, it was shown in a clinical study [20], [21] that treatment alternation leads to significantly less virological failure than conventional HAART, when compliance is imperfect but identical between the two study arms. However, since the study in [20], [21] is not identical to the treatment strategy presented herein, a clinical study should be performed to fully investigate the potential of the proposed induction-maintenance strategy. Ideally, this prospective randomized trial could evaluate the time to virological failure in patients taking a single unchanged regimen and patients on induction-maintenance regimens. Importantly, the trial should be designed to evaluate whether the induction maintenance strategy affects the durability of second- and third line regimens. The presence and relative frequency of viral minority populations as well as their mutational patterns could be monitored by analyzing proviral DNA from circulating T-cells using, e.g., next-generation sequencing. This data could serve to validate our mathematical model.

Based on a recent, successful pre-exposure prophylaxis (PrEP) trial, where emtricitabine (FTC) + tenofovir (TDF) were given to high-risk individuals [53], it could be envisioned that PrEP is used more broadly. One risk with such a strategy is the selection of FTC/TDF resistance, which occurred in both subjects with acute HIV infection at enrolment in the PrEP trial [53]. Furthermore, there is a high risk for the selection of drug resistance, if subjects get infected despite PrEP (e.g. due to low adherence; 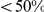 in the PrEP trial [53]). While FTC/TDF is a core component of first-line HAART, the long-term epidemiological consequences of drug-resistance selection are of utmost importance. One interesting question is whether the proposed induction-maintenance therapy can re-sensitize those subjects towards FTC/TDF treatment, who had become infected with HIV despite PrEP. While a thorough analysis of this question is beyond the scope of the current article, related scenarios are frequently encountered in the context of prevention of mother-to-child transmission (MTCT) programs, when short-course intrapartum nevirapine is used. In the MTCT context, protease-inhibitor-based induction therapy has been used for the re-sensitization of pre-exposed children towards nevirapine [54]. Further analysis, however, is required to elucidate the potential of induction-maintenance strategies for re-sensitization of pre-exposed HIV infected individuals.

## Materials and Methods

### Model Equations

The virus dynamics model (Fig. 1) comprises T-cells, macrophages, free non-infectious virus (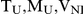, respectively), free infectious virus of mutant strain 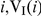, and five types of infected cells belonging to mutant strain 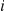: infected T-cells and macrophages *prior* to proviral genomic integration (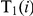 and 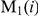, respectively) and infected T-cells and macrophages *after* proviral genomic integration (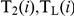 and 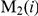, respectively). The latently infected cell type 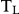 does not express viral genes, but can become activated with rate 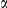, transforming this cell into a virus producing post-integration infected T-cell 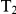. The average rates of change of the different species are given by the following system of ODEs:
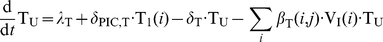


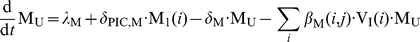


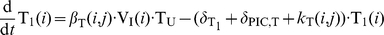


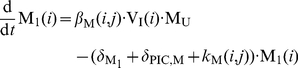
(3)

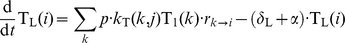


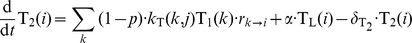


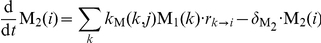


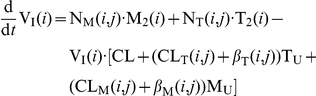


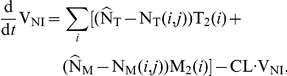
where 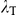 and 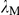 are the birth rates of uninfected T-cells and macrophages, and 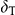 and 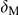 denote their death rate constants. The parameters 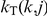 and 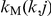 are the integration rate constants of mutant strain 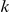 under treatment 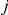. The parameters 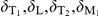 and 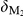 are the death rate constants of 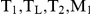 and 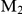 cells, respectively. The free virus (infectious and non-infectious) gets cleared by the immune system with rate constant 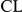. The parameters 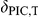 and 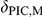 refer to the intracellular degradation of essential components of the pre-integration complex, e.g., by the host cell proteasome within early infected T-cells and macrophages, respectively. 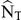 and 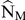 denote the total number of released infectious and non-infectious virus from late infected T-cells and macrophages of mutant strain 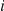, and 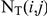 and 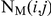 are the rates of release of infective virus under treatment 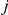. The parameters 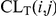 and 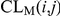 denote the clearance of mutant virus 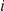 through unsuccessful infection of T-cells and macrophages, respectively [23], and the parameters 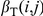 and 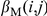 denote the successful infection rate constants of mutant virus 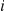 under treatment 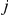 for T-cells and macrophages, respectively. In our model, T-cells can become latently infected 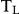 with probability 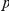. Latent infected cells can undergo apoptosis with rate 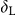 and can become activated with rate 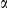. Activation of latent cells by antigen- or other activating stimuli triggers the production of viral building blocks via positive feedback loops [55], [56] in the late replication cycle of HIV, which turns the cell into a virus producing cell 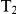 that becomes susceptible to HIV-related cytopathic effects and destruction by the immune system.

The parameter 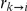 denotes the probability to mutate from strain 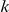 to strain 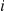 and is defined by
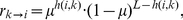
(4)where 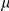 denotes the point mutation probability per base and reverse transcription process (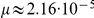 [42]), 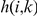 denotes the hamming distance between strain 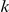 and strain 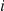, and 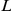 is the total number of different positions that are considered in our model (here, 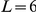 point mutations). In total, the model includes 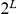 different viral strains 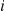 that contain point mutations in any pattern of the modelled 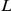 possible mutations. The phenotype of each mutant strain 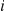 is modelled by introducing a selective disadvantage 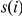, which denotes the loss of functionality (e.g., in the activity of some viral enzyme that is affected by the mutation) relative to the wildtype, and a strain specific inhibitory activity 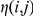 of treatment 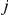 against the mutant strain 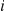. For example, the strain specific infection rate 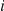 under a certain treatment 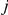 is given by 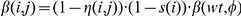, where 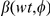 denotes the infection rate constant of the wildtype 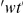 in the absence of drug 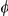 (parameters listed in Table 1). The strain-specific specific inhibitory activity is calculated via 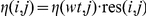, where the efficacy of the drugs against the wildtype 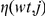 is generally stated in the corresponding tables and figures (Fig. 2, Fig. 5 and Table 2) and the resistance of a particular mutant 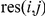 was either set to 1 (100% susceptible) or 0.01 (99% resistant), if the particular mutant 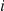
conferred resistance to the particular drug 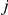.

All parameter values have been chosen according to previous studies (see Table 1). The particular viral decay dynamics after application of distinct drug classes were validated in [23]. The model (Fig. 1) with above described parameters reproduces an average frequency of latently infected cells of 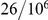 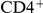 cells (reference range: 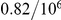 – 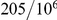 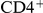 cells [2], [7], [30], [31]), a total of 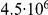 latently infected cells (reference: 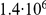 [7]), with a halflife of 20.6 month (average of [2], [57]–[60]: 21 month) and a plasma viremia of 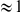 HIV RNA/mL [61] from the latent reservoir.

### Realization and Implementation of the Model

The overall virus dynamics in our model comprise different viral strains with copy numbers that can vary over several orders of magnitude. For this reason we have chosen a hybrid (stochastic-deterministic) setting for numerical simulation. This approach (i) takes into account stochastic fluctuations in the slow reaction processes; and (ii) reduces the computational costs for the simulation of the fast (deterministic) system dynamics. We used the direct hybrid method proposed in [25], where we treated elementary reactions 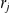 as discrete stochastic processes whenever their propensity function 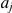 or the quantity of at least one of their reactants was below a threshold of 20. All other reactions were approximated as continuous deterministic processes. Elementary reactions 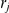 with propensity functions 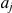 and their respective net changes 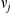 can be deduced from eqs. (3). For example, the term 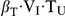 denotes the infection reaction of T-cells by infectious virus. The propensity function of this reaction is 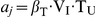. This reaction changes the species levels as follows: one 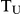 cell and one 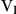 virus get consumed (the term is once subtracted from each corresponding ODE), and one 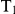 cell is produced (the term is once added to the ODE of 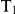).

In brief, the hybrid method comprises the following algorithmic workflow:

1. Set initial time 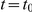 and initial number of molecules 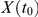.
2. Generate two uniformly distributed pseudo-random variables 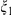 and 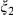 on the open unit interval 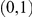 and determine the partitioning of reactions into deterministic and stochastic subsets 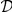 and 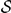, respectively. The latter is realized by comparing the actual propensity and the reactant levels of every reaction with pre-specified thresholds. If one value is below the thresholds, a reaction is included in the stochastic subset 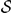, otherwise it is put in the deterministic subset 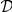.
3. Set 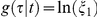 and solve the ODE system for the deterministic part of the system starting at time 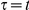


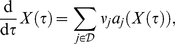
(5)together with
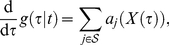
(6)until time 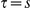 such that 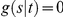.
4. Take the integer 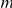 satisfying
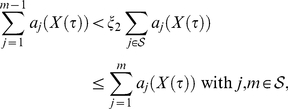
(7)in order to determine the stochastic reaction 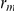 to be performed.
5. Update 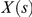 according to reaction 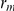, hence set 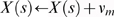.
6. Set 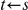, and stop the procedure if the final time is reached. Otherwise go to Step (2).

The above algorithmic scheme requires the use of numerical integrators that allow to stop integration in step (3) when a stochastic reaction event is detected at a time 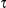 where 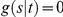. The utilized integrator is based on numerical differentiation formulas [62], and uses strategies for event detection and error- and step size control comparable to ode15s in Matlab [63]. To generate the data for Fig. 4, we performed 12000 hybrid simulations in total. With realization start (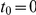) the effects of drug treatment were simulated, until 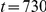 days was reached. Every numerical calculation was computed with a relative error tolerance of 10^−6^ and an absolute error tolerance of 10^−9^. Our simulation code is provided in Source Code S1–S6.

### Reproductive Numbers

For the model above (eq. (3)), the reproductive numbers, which indicate the expected number of offspring in the next generation, are defined as follows: the reproductive number 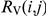 of a single virus of strain 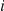 under treatment 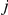 is given by 
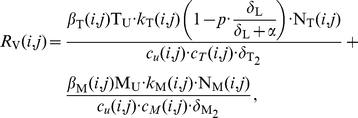
with constants
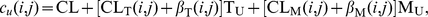


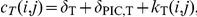


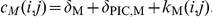



Since infected cells are also pathogens, which can lead to a rebound of the disease even in the absence of any virus, we also determined their basic reproductive numbers under a given treatment 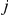. The basic reproductive numbers 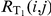 and 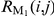 of the infectious stages 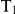 and 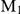, associated with the viral strain 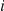, are given by 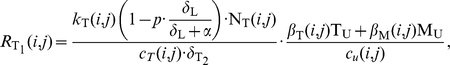


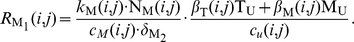
Finally, the reproductive numbers 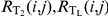 and 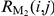 of the infectious stages 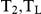 and 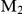, associated with the viral strain 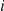, are given by 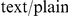


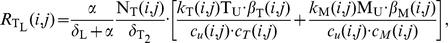


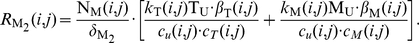



### Reproductive Capacity

We have previously introduced the reproductive capacity 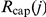 [23], which can be interpreted as the expected total number of infectious offspring that the infection produces in one round of replication under a certain treatment 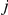, given the current state of the infection. In this article, we utilize the reproductive capacity in order to get individual treatment switching times (see eq. (1), main article), which are displayed in Fig. 3. The reproductive capacity of the entire quasi-species ensemble under treatment 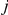 is defined as the weighted sum of the basic reproductive numbers of all pathogenic stages of mutant strain 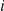, i.e., free virus, infected T-cells and infected macrophages, weighted by the abundance of the corresponding pathogenic stage [23]:
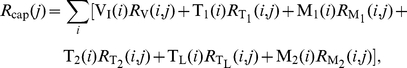
where 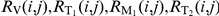 and 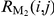 are the strain-specific reproductive numbers of the different infective compartments (see previous sections).

## Supporting Information

Figure S1
**Time and probability of virological failure depends on pool-size of archived drug-resistant virus.** A: The median time until virological failure, in relation to the number of fully-resistant archived virus (fully  =  resistant against all drugs in the triple-drug combination). B: Probability that virological failure occurs within two years after initiation of HAART therapy as a function of the number of fully-resistant archived virus. 500 stochastic-deterministic runs were performed for each pool size of the latently infected drug-resistant reservoir. Parameter values used: 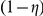 = 0.75, 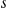 = 0.8.(PDF)

Table S1
**Determination of relevant parameter space for further investigation.** We assessed virological failure rates after one year of triple drug therapy for varying values of efficacy 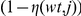 of drug 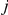 against the wildtype 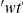 and selective disadvantage per mutation 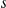. All other parameters have been taken from Table 1. A parameter combination (in terms of 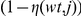 and 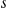) was considered relevant, if it produced realistic failure rates after one year of therapy [29]. Confidence ranges are indicated in brackets and were calculated using Greenwood's formula. Each condition has been evaluated by 100 stochastic deterministic simulations.(PDF)

Source Code S1 The File ‘HAART.m’ can be used to simulate the kinetics of HIV after application of conventional HAART treatment in MATLAB.
(M) Click here for additional data file.

Source Code S2 The File ‘HIVmodel.m’ builds the original HIV model used throughout the manuscript for use in MATLAB.
(M) Click here for additional data file.

Source Code S3 The File ‘InductionMaintenance.m’ can be used to simulate the kinetics of HIV after under the proposed induction-maintenance therapy in MATLAB.
(M) Click here for additional data file.

Source Code S4 The File ‘PatientMonitoring.m’ contains the MATLAB implementation of routine patient monitoring.
(M) Click here for additional data file.

Source Code S5 The File ‘ReadMeFirst.txt’ Contains a description of all supplied source code files, contact details, information on runtime and execution and a copy of the GNU public license.
(TXT)

Source Code S6 The File ‘SpeciesLevelsIndices.pdf’ contains an interpretation of the output generated by executing the provided MATLAB Source Code Files (Source Code S1–S4).
(PDF)
