# Supplementary figures and images for: HIV Quasispecies Dynamics during Pro-Active Treatment Switching: Impact on Multi-Drug Resistance and Resistance Archiving in Latent Reservoirs
Source: PLoS One. 2011 Mar 24;6(3):e18204. doi: 10.1371/journal.pone.0018204 (PMC3063788; doi:10.1371/journal.pone.0018204)

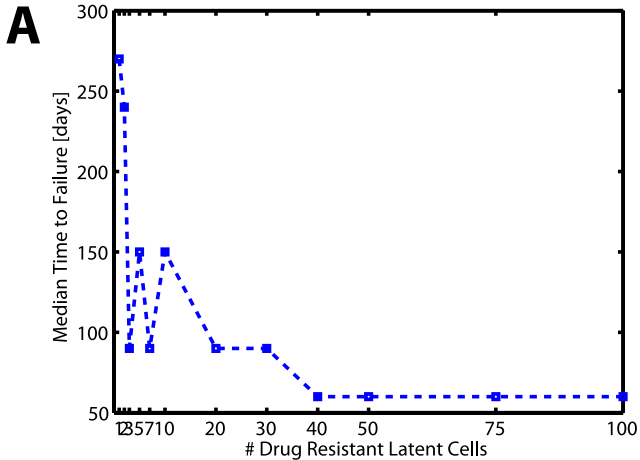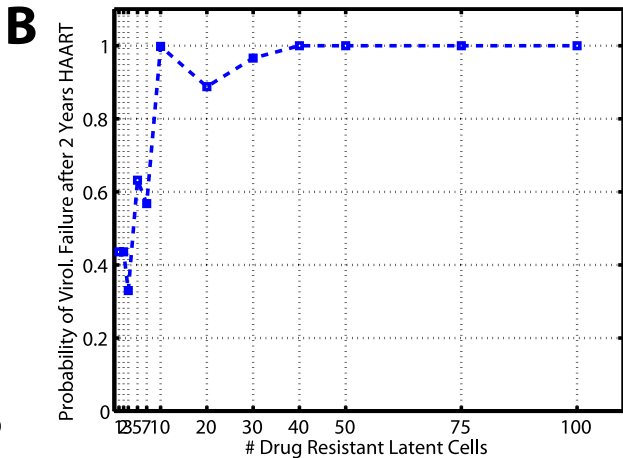

Supplement: Figure S1 — Time and probability of virological failure depends on pool-size of archived drug-resistant virus. A: The median time until virological failure, in relation to the number of fully-resistant archived virus (fully = resistant against all drugs in the triple-drug combination). B: Probability that virological failure occurs within two years after initiation of HAART therapy as a function of the number of fully-resistant archived virus. 500 stochastic-deterministic runs were performed for each pool size of the latently infected drug-resistant reservoir. Parameter values used: = 0.75, = 0.8. (PDF) [file pone.0018204.s001.pdf]
