# Supplementary material for: HIV Quasispecies Dynamics during Pro-Active Treatment Switching: Impact on Multi-Drug Resistance and Resistance Archiving in Latent Reservoirs
Source: PLoS One. 2011 Mar 24;6(3):e18204. doi: 10.1371/journal.pone.0018204 (PMC3063788; doi:10.1371/journal.pone.0018204)
Supplement: Table S1 — Determination of relevant parameter space for further investigation. We assessed virological failure rates after one year of triple drug therapy for varying values of efficacy of drug against the wildtype and selective disadvantage per mutation . All other parameters have been taken from Table 1. A parameter combination (in terms of and ) was considered relevant, if it produced realistic failure rates after one year of therapy [29]. Confidence ranges are indicated in brackets and were calculated using Greenwood's formula. Each condition has been evaluated by 100 stochastic deterministic simulations. (PDF) [file pone.0018204.s002.pdf]

Table S1. Determination of relevant parameter space for further investigation.

Par. set	$(1-\eta)$	s	failure rate	relevant
R1	0.7	0.3	22% (±6.4)	yes
R2	0.7	0.25	22% (±6.4)	yes
R3	0.7	0.2	53% (±9.8)	yes
R4	0.75	0.25	3% (±3.3)	yes
R5	0.75	0.2	18% (±7.5)	yes
R6	0.8	0.2	2% (±2.7)	yes
R7	0.8	0.15	21% (±8)	yes
R8	0.8	0.1	51% (±9.8)	yes
R9	0.85	0.15	5% (±4.3)	yes
R10	0.85	0.1	14% (±6.8)	yes
R11	0.85	0.05	64% (±9.4)	yes
R12	0.9	0.05	2% (±2.7)	yes
N13	0.7	0.15	90% (±5.9)	no
N14	0.7	0.1	100% (-)	no
N15	0.7	0.05	100% (-)	no
N16	0.75	0.3	1% (±2)	no
N17	0.75	0.15	68% (±9.14)	no
N18	0.75	0.1	99% (±1)	no
N19	0.75	0.05	100% (-)	no
N20	0.8	0.3	0% (-)	no
N21	0.8	0.25	0% (-)	no
N22	0.8	0.05	100% (-)	no
N23	0.85	0.3	0% (-)	no
N24	0.85	0.25	0% (-)	no
N25	0.85	0.2	0% (-)	no
N26	0.9	0.3	0% (-)	no
N27	0.9	0.25	0% (-)	no
N28	0.9	0.2	0% (-)	no
N29	0.9	0.15	0% (-)	no
N30	0.9	0.1	0% (-)	no
N31	0.95	0.3	0% (-)	no
N32	0.95	0.25	0% (-)	no
N33	0.95	0.2	0% (-)	no
N34	0.95	0.15	0% (-)	no
N35	0.95	0.1	0% (-)	no
N36	0.95	0.05	0% (-)	no

We assessed virological failure rates after one year of triple drug therapy for varying values of efficacy $\big(1-\eta(wt,j)\big)$ of drug j against the wildtype wt and selective disadvantage per mutation s. All other parameters have been taken from Table 1. A parameter combination (in terms of $\big(1-\eta(wt,j)\big)$ and s) was considered relevant, if it produced realistic failure rates after one year of therapy [1]. Confidence ranges are indicated in brackets and were calculated using Greenwood's formula. Each condition has been evaluated by 100 stochastic deterministic simulations.

References

1. Riddler SA, Haubrich R, DiRienzo AG, Peeples L, Powderly WG, et al. (2008) Class-sparing regimens for initial treatment of HIV-1 infection. N Engl J Med 358: 2095–2106.
