# Supplementary material for: HIV Quasispecies Dynamics during Pro-Active Treatment Switching: Impact on Multi-Drug Resistance and Resistance Archiving in Latent Reservoirs
Source: PLoS One. 2011 Mar 24;6(3):e18204. doi: 10.1371/journal.pone.0018204 (PMC3063788; doi:10.1371/journal.pone.0018204)
Supplement: Source Code S6 — The File ‘SpeciesLevelsIndices.pdf’ contains an interpretation of the output generated by executing the provided MATLAB Source Code Files (Source Code S1–S4). (PDF) [file pone.0018204.s008.pdf]

Index		wt	Mut1	Mut2	Mut12
1	TU				
2	MU				
3		T1(wt)			
4		T2(wt)			
5		TL(wt)			
6		M1(wt)			
7		M2(wt)			
8		VI(wt)			
9			T1(mut1)		
10			T2(mut1)		
11			TL(mut1)		
12			M1(mut1)		
13			M2(mut1)		
14			VI(mut1)		
15				T1(mut2)	
16				T2(mut2)	
17				TL(mut2)	
18				M1(mut2)	
19				M2(mut2)	
20				VI(mut2)	
21					T1(mut12)
22					T2(mut12)
23					TL(mut12)
24					M1(mut12)
25					M2(mut12)
26					VI(mut12)
27	VNI				
